# Alcohol dehydrogenases from *Kluyveromyces marxianus*: heterologous expression in *Escherichia coli* and biochemical characterization

**DOI:** 10.1186/1472-6750-14-45

**Published:** 2014-05-21

**Authors:** Jing-juan Liang, Mei-ling Zhang, Meng Ding, Zhi-mao Mai, San-xing Wu, Yue Du, Jia-xun Feng

**Affiliations:** 1 College of Life Science and Technology, Guangxi University, 100 Daxue Road, Nanning, Guangxi 530004, P. R. China; 2 State Key Laboratory for Conservation and Utilization of Subtropical Agro-bioresources, Guangxi University, 100 Daxue Road, Nanning, Guangxi 530004, P. R. China

**Keywords:** Alcohol dehydrogenase, Characterization, Expression, Gene cloning, *Kluyveromyces marxianus*

## Abstract

**Background:**

*Kluyveromyces marxianus* has recently become a species of interest for ethanol production since it can produce ethanol at high temperature and on a wide variety of substrates. However, the reason why this yeast can produce ethanol at high temperature is largely unknown.

**Results:**

The ethanol fermentation capability of *K. marxianus* GX-UN120 at 40°С was found to be the same as that of *Saccharomyces cerevisiae* at 34°С. Zymogram analysis showed that alcohol dehydrogenase 1 (KmAdh1) was largely induced during ethanol production, KmAdh4 was constitutively expressed at a lower level and KmAdh2 and KmAdh3 were almost undetectable. The genes encoding the four alcohol dehydrogenases (ADHs) were cloned from strain GX-UN120. Each *KmADH* was expressed in *Escherichia coli* and each recombinant protein was digested with enterokinase to remove the fusion protein. The optimum pH of the purified recombinant KmAdh1 was 8.0 and that of KmAdh2, KmAdh3 and KmAdh4 was 7.0. The optimum temperatures of KmAdh1, KmAdh2, KmAdh3 and KmAdh4 were 50, 45, 55 and 45°C, respectively. The *K*_m_ values of the recombinant KmAdh1 and KmAdh2 were 4.0 and 1.2 mM for acetaldehyde and 39.7 and 49.5 mM for ethanol, respectively. The *V*_max_ values of the recombinant KmAdh1 and KmAdh2 were 114.9 and 21.6 μmol min^-1^ mg^-1^ for acetaldehyde and 57.5 and 1.8 μmol min^-1^ mg^-1^ for ethanol, respectively. KmAdh3 and KmAdh4 catalyze the oxidation reaction of ethanol to acetaldehyde but not the reduction reaction of acetaldehyde to ethanol, and the *K*_*m*_ values of the recombinant KmAdh3 and KmAdh4 were 26.0 and 17.0 mM for ethanol, respectively. The *V*_max_ values of the recombinant KmAdh3 and KmAdh4 were 12.8 and 56.2 μmol min^-1^ mg^-1^ for ethanol, respectively.

**Conclusion:**

These data in this study collectively indicate that KmAdh1 is the primary ADH responsible for the production of ethanol from the reduction of acetaldehyde in *K. marxianus*. The relatively high optimum temperature of KmAdh1 may partially explain the ability of *K. marxianus* to produce ethanol at high temperature. Understanding the biochemical characteristics of KmAdhs will enhance our fundamental knowledge of the metabolism of ethanol fermentation in *K. marxianus*.

## Background

*Kluyveromyces marxianus* is a sister species to the better-known *K. lactis*[1]. A large number of studies on *K. lactis* have mainly focused on its lactose metabolism and use as a model for non-conventional yeasts [2]. In contrast, scientific literature about the fundamental aspects of *K. marxianus* is relatively scarce [1]. Recently, *K. marxianus* has gained increasing attention since some of its traits are desirable for biotechnological applications. These traits include the fastest growth rate of any eukaryotic microbe, thermotolerance, secretion of native enzymes such as inulinase, β-galactosidase and pectinase, and production of ethanol [1,3].

*K. marxianus* is now being investigated as an alternative to *Saccharomyces cerevisiae* for ethanol production, especially in simultaneous saccharification and fermentation (SSF) or simultaneous saccharification and co-fermentation (SSCF) processes, since it can produce ethanol at higher temperatures and on a wider variety of substrates including xylose [3-5]. It has been reported to be able to grow at 45°С and even 52°С and to produce ethanol at temperatures above 40°C [4,6,7]. *S. cerevisiae*, in contrast, is unable to ferment xylose and has an optimum growth temperature ranging from 30 to 34°С [8]. The enzymatic hydrolysis during SSF or SSCF processes is usually conducted at approximately 50°C, and the products formed during the hydrolysis step in SSCF include hexoses and pentoses. The traits of *K. marxianus* make it suitable for use in SSCF processes involving cellulosic biomass [9,10].

Yeast alcohol dehydrogenase (ADH) catalyzes the final metabolic step in ethanol fermentation, and thus plays an important role. The ADH systems of *S. cerevisiae* and *K. lactis* were studied extensively and seven *ScADH* genes (*ScADH1* to *ScADH7*) and four *KlADH* genes (*KlADH1* to *KlADH4*) have reportedly been cloned [11-14]. There are only a few scientific papers on the ADH systems of *K. marxianus*. Recently, the complete genome sequence of *K. marxianus* var. *marxianus* KCTC 17555 was determined and four ADH-encoding genes were annotated in the genome [15]. Two genes, *KmADH1* and *KmADH2*, were cloned from *K. marxianus* ATCC 12424, while other two genes, *KmADH3* and *KmADH4*, were cloned from *K. marxianus* DMKU 3–1042 [12,16-18]. However, heterologous expression of the four genes and the biochemical properties of the KmAdhs have not been reported yet.

The *K. marxianus* GX-UN120 strain obtained in our laboratory is an excellent ethanol producer at high temperature and produced 69 g/L of ethanol when fermenting 150 g/L of glucose at 40°C [19]. Determining the biochemical characteristics of the ADHs of GX-UN120 will help to explain why it can produce high levels of ethanol at high temperature. In the present study, the genes encoding the four KmAdhs of GX-UN120 were cloned and individually overexpressed in *E. coli*, and the biochemical characteristics of each purified KmAdh were investigated. Understanding the biochemical characteristics of the KmAdhs of *K. marxianus* will enhance our fundamental knowledge of the ADH systems and the metabolism of ethanol fermentation in *K. marxianus*.

## Results

### Growth and ethanol fermentation characteristics of *K. marxianus* GX-UN120

**Figure 1:**
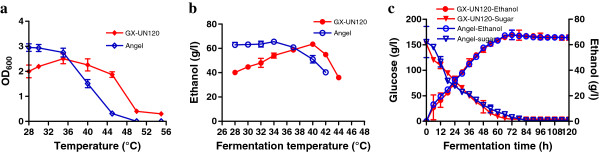
**The growth and ethanol fermentation characteristics of *K. marxianus *GX-UN120 and *S. cerevisiae *Angel.** Exponential phase cultures of GX-UN120 and Angel were used to inoculate YPD medium containing 20 g/L glucose to a final OD_600_ of 0.2 or fermentation medium containing 150 g/L glucose with 10% of the inoculum. **(a)** Growth in YPD medium containing 20 g/L glucose for 24 h without shaking. **(b)** Ethanol fermentation was carried out in 150 g/L glucose at different temperatures for 72 h without shaking. **(c)** The time course of ethanol fermentation was recorded in 150 g/L glucose at 40°C (GX-UN120) or 34°C (Angel) without shaking. Experiments were performed in triplicate and the results are given as mean values with error bars indicating standard deviations.

*K. marxianus* GX-UN120 is an excellent ethanol-producing mutant strain that was converted from the wild-type strain GX-15 by alternately treatment with UV irradiation and NTG for two cycles. When fermenting 150 g/l of glucose, the ethanol yield of GX-UN120 was 69 g/l which was 20% higher than that of GX-15. However, the ADH activity of GX-UN120 was not significantly higher than that of GX-15 [19]. The nucleotide sequence of *KmADH1* in GX-UN120 (KF678864) was not different to that in GX-15 (JF709970). The growth and ethanol fermentation characteristics of GX-UN120 were determined compared with those of *S. cerevisiae* Angel, which is a commercial ethanol producer in China. The optimum temperatures for growth and ethanol fermentation of GX-UN120 were 35-40°С and 40°С, respectively, whereas that of Angel was 28-34°С. GX-UN120 grew well even at 45°С, whereas Angel was not able to grow when the temperature was over 45°С (Figure 1a and b). The time courses for ethanol formation in 150 g/L glucose by GX-UN120 at 40°С and Angel at 34°С are shown in Figure 1c. The time taken for GX-UN120 to completely consume the glucose and reach its maximum ethanol yield was the same as that for Angel. Both yeasts consumed the glucose completely within 72 h. At that time the maximum ethanol concentration and ethanol yield coefficient of GX-UN120 were 67.6 g/L and 0.45 g/g, respectively, and those of Angel were 67.7 g/L and 0.45 g/g, respectively.

### Analysis of the expression of *KmADHs* in *K. marxianus* GX-UN120

**Figure 2:**
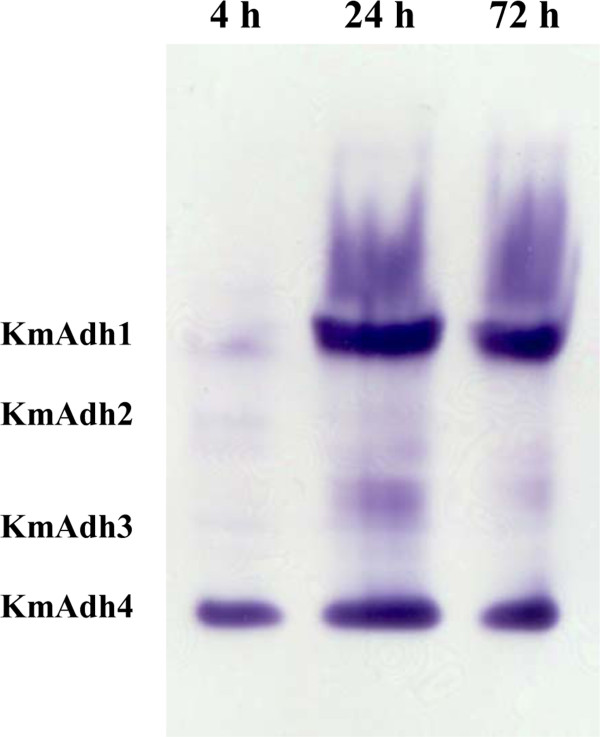
**Zymogram analysis of ADH isozymes from GX-UN120 during ethanol fermentation.** Fermentation was performed in YPD containing 150 g/L glucose at 40°C. Cells were harvested from the broth at the lag (4 h), exponential (24 h) and stationary phases (72 h) and disrupted by grinding on ice. The cell extracts were separated on 7.5% polyacrylamide gel and the gels were stained for ADH activity with ethanol as the substrate.

The translational levels of *KmADH* genes in GX-UN120 were determined through the analysis of zymograms of the ADH isozymes at different fermentation phases (the lag, exponential and stationary phases) in YPD containing 150 g/L of glucose (Figure 2). The results indicated that *KmADH1* was weakly expressed at the lag phase and largely expressed at the exponential phase, and its expression level decreased at the stationary phase. *KmADH4* was constitutively expressed during all phases. The expression levels of *KmADH2* and *KmADH3* were not detectable.

### Cloning and sequence analysis of the genes encoding the four KmADHs from *K. marxianus* GX-UN120

The four genes encoding ADHs, *KmADH1*, *KmADH2*, *KmADH3* and *KmADH4*, were cloned from GX-UN120 and sequenced. The open reading frames (ORFs) of the four ADH genes were, respectively, 1047, 1047, 1128 and 1140 bp and the deduced amino acid sequences were 348, 348, 375 and 379 amino acids, respectively. The deduced amino acid sequences of the four KmAdhs from GX-UN120 shared 98% to 99% identity with the corresponding four genes of ATCC 12424 and more than 80% identity with the ADHs of *K. lactis*, *K. wickerhamii*, *S. cerevisiae*, *S. carlsbergensis*, *S. kluyveri*, *S. pastorianus* and *Hansenula polymorpha*[11-14,16,17,20-23]. There are five amino acid residues difference in the deduced amino acid sequence of KmADH1 in GX-UN120 and KmADH1 in ATCC 12424, they are N15H, G239V, T328S, S334V and I339V. In KmADH2, the different amino acid residues are H315N and I338V. In KmADH3, the different amino acid residues are E233D and Q240E. In KmADH4, the different amino acid residues are N268S, V360I and S378A. All these amino acid residues are not in the groups directly involved in catalysis.

**Figure 3:**
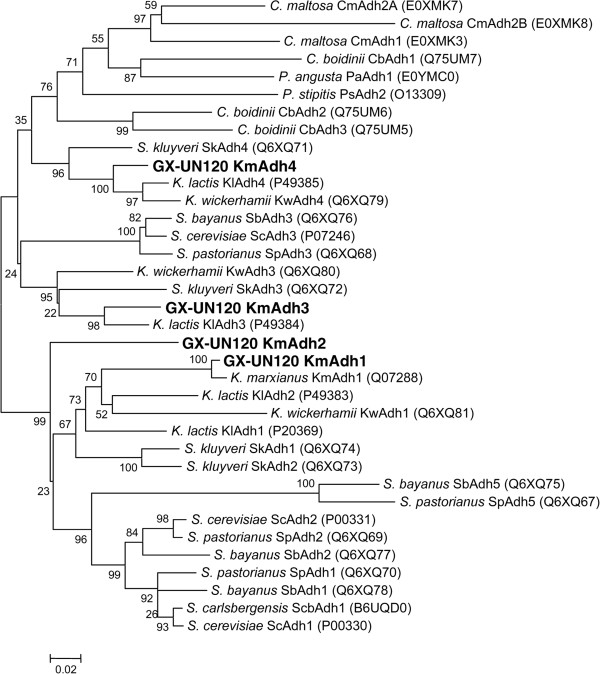
**Phylogenetic analysis of KmAdhs of GX-UN120.** The sequences were aligned to generate an unrooted phylogenetic tree with MEGA 4.0 using the neighbor-joining method. Branch support values from 1000 bootstrap replications are presented beside each node and a Poisson correction was carried out. GenBank accession numbers are shown in brackets after each enzyme name. All proteins included in the analysis were enzymatically characterized as alcohol dehydrogenases. References are listed in Additional file 1: Table S1 in the supplementary materials.

The phylogenetic analysis of the amino acid sequences of the KmAdhs and yeast ADHs (Additional file 1: Table S1) in Figure 3 reveals that KmAdh1 of GX-UN120 is closely grouped with Adh1 from *K. marxianus* ATCC 12424, *K. marxianus* DMKU3-1042 and *K. wickerhamii* and Adh2 from *K. lactis*, and KmAdh2 is grouped with Adh2 from *K. marxianus* ATCC 12424, whereas KmAdh3 and KmAdh4 are closely grouped with Adh3 and Adh4 from *K. marxianus* ATCC 12424, *K. marxianus* DMKU3-1042, *K. wickerhamii* and *K. lactis*.

**Figure 4:**
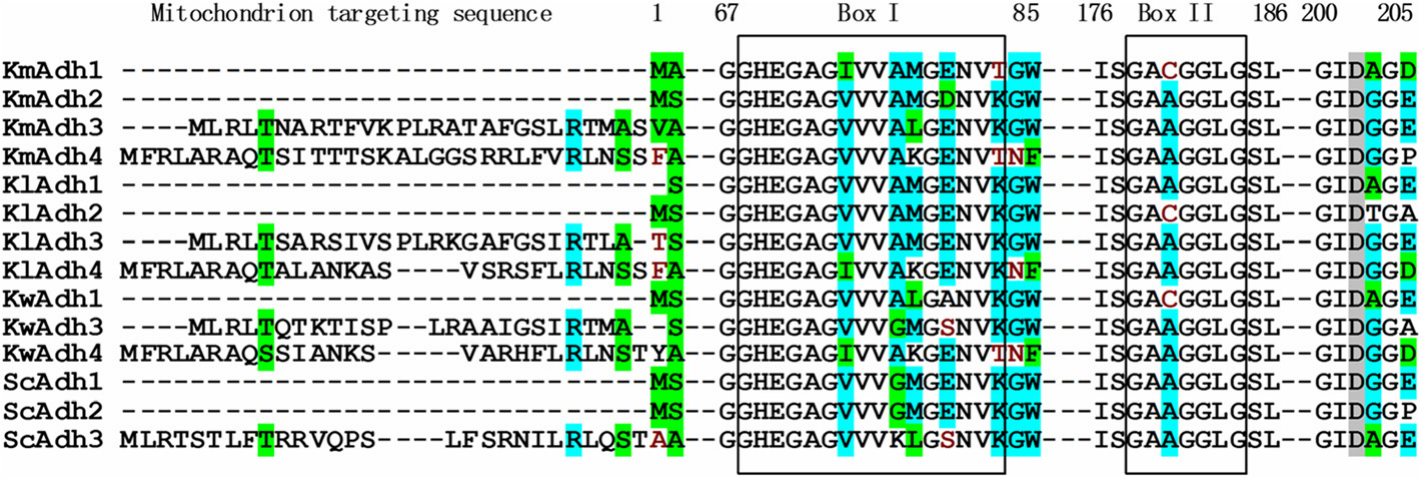
**Alignment of the conserved amino acid residues and structurally conserved regions of the KmAdhs.** Alignment was done using the Vector NTI program. The protein codes correspond to those listed in Additional file 1: Table S1 in the supplementary material. Asp residues in deep grey determine the specificity for NAD^+^. Residues in box I and box II indicate Zn^2+^-binding and NAD^+^-binding moieties, respectively. KmAdh, alcohol dehydrogenase from *K. marxianus*; KlAdh, alcohol dehydrogenase from *K. lactis*; KwAdh, alcohol dehydrogenase from *K. wickerhamii*; ScAdh, alcohol dehydrogenase from *S. cerevisiae*.

The multiple amino acid sequence alignments of KmAdhs with ADHs from other yeasts (Additional file 1: Table S1) reveal that KmAdh1 and KmAdh2 do not possess but KmAdh3 and KmAdh4 do possess the N-terminal mitochondrion targeting sequence (Figure 4). These results indicate that KmAdh1 and KmAdh2 are cytoplasmic ADHs, whereas KmAdh3 and KmAdh4 are mitochondrial ADHs. Several conserved motifs of the microbial group I ADHs were found in the KmADHs, including Asp^202^ in KmAdh1 and KmAdh2, Asp^229^ in KmAdh3 and Asp^233^ in KmAdh4, which determine the NAD^+^ specificity. This suggests that KmAdhs are NAD-dependent ADHs similar to the ADHs of other yeasts [24]. The NAD^+^-binding motifs of the KmAdhs are GAG/CGGLG (BoxII), similar to those in ADHs of other yeasts [12,21,25]. Zn^2+^-binding residues, which are known to be essential for enzyme catalytic activity and structure, and the Zn^2+^-binding consensuses were also found (Box I).

### Expression and purification of the recombinant KmADHs

**Figure 5:**
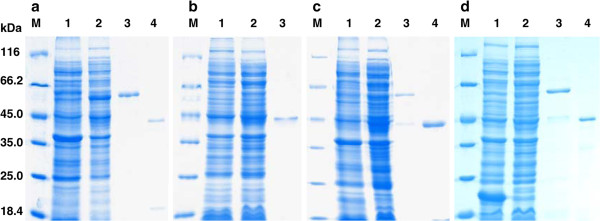
**Electrophoresis of KmAdhs from *K. marxianus *GX-UN120.** SDS-PAGE analysis on 10% polyacrylamide gel stained with Coomassie light blue. **a**, KmAdh1; **b**, KmAdh2; **c**, KmAdh3; **d**, KmAdh4. Lane M, protein molecular weight markers (116.0, 66.2, 45.0, 35.0 25.0 and 18.4 kDa). Lane 1, proteins of *E. coli* Rosetta DE3 harboring the empty plasmid pET-32a(+) in **a**, **c**, **d** and empty pET-30a(+) in b; lane 2, proteins of *E. coli* Rosetta DE3 harboring the plasmids pET-32a(+)-KmADH1, pET-32a(+)-KmADH3 and pET-32a(+)-KmADH4 in **a**, **c** and **d** and pET-30a(+)-KmADH2 in **b**; lane 3, the purified recombinant KmAdhs fusion proteins; lane 4, the purified KmAdhs. The recombinant fusion proteins and proteins after digestion with enterokinase light chain were purified with Co–NTA chromatography.

The four cloned ADH genes were expressed in *E. coli* Rosetta DE3. The recombinant KmAdhs were purified and then digested with enterokinase light chain. A clear, single band for each of the KmAdh1, KmAdh2, KmAdh3 and KmAdh4 fusion proteins corresponding to about 58, 59, 49 and 60 kDa, respectively, was clearly seen on SDS-PAGE (Figure 5). The purified recombinant KmAdh1, KmAdh3 and KmAdh4, after digestion with enterokinase light chain, had molecular masses corresponding to about 48, 48 and 49 kDa, respectively, as observed by SDS-PAGE (Figure 5). The purified recombinant KmAdh2 had a molecular mass of about 48 kDa (data not shown). The molecular masses of the purified KmAdh1, KmAdh2, KmAdh3 and KmAdh4, as determined by HPGPC, were about 190, 190, 190 and 200 kDa, respectively (data not shown). These results indicate that each of the four KmAdhs of GX-UN120 is active as a homotetramer.

### Biochemical characterization of the recombinant KmAdhs

**Figure 6:**
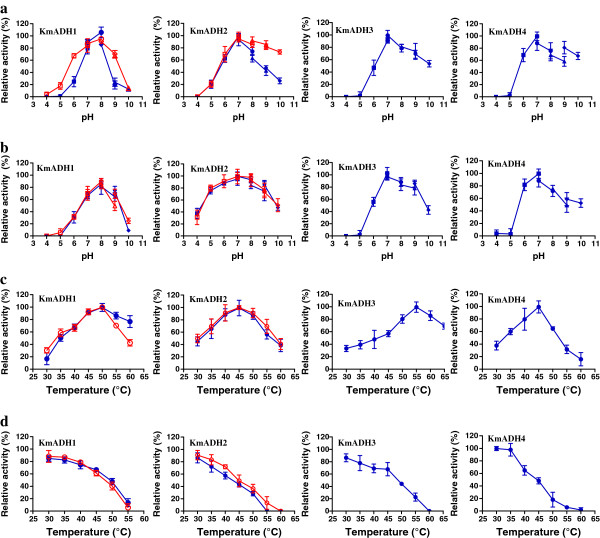
**Effects of pH and temperature on enzyme activities of KmAdhs. a** Determination of the optimal pH of the KmAdhs. Enzyme assays were performed at the indicated pH at 40°C using ethanol (*closed symbols*) and acetaldehyde (*open symbols*) as substrates. *squares*, 50 mM citrate-phosphate buffer (pH 4.0-7.0); *circles*, 50 mM sodium-phosphate buffer (pH 6.0-8.0); *triangles*, 50 mM Tris–HCl buffer (pH 8.0-9.0); *diamonds*, 50 mM glycine-NaOH buffer (pH 9.0-10.0). **b** pH stability of the KmAdhs. Enzyme activity was measured under optimal conditions (50 mM sodium-phosphate buffer of pH 7.0, 40°C) after the enzyme was incubated in the indicated buffers at 4°C for 24 h. **c** Determination of the optimal temperatures of the KmAdhs. Activity was measured at pH 7.0 (50 mM sodium-phosphate buffer) at the indicated temperatures. **d** Thermal stability of KmAdhs. Enzyme activity was measured under optimal conditions (50 mM sodium-phosphate buffer of pH 7.0, 40°C) after the enzyme had been incubated at the indicated temperature for 30 min. The error bars represent the standard deviations of triplicate measurements.

The purified recombinant KmAdhs without tags were used for the characterization of enzymatic properties. The specific ADH activities of KmAdh1 and KmAdh2 were 102.5 and 19.3 U/mg for acetaldehyde and 47.5 and 1.5 U/mg for ethanol, respectively. Those of KmAdh3 and KmAdh4 were, respectively, 10.1 and 46.0 U/mg for ethanol and no activities were detected for acetaldehyde. ADH activities were investigated with NAD^+^ or NADP^+^ and NADH or NADPH as cofactors to determine cofactor preference. The specific activities of KmAdhs with NAD^+^ and NADH were 70–80 times and 50–60 times higher than those with NADP^+^ and NADPH, respectively. These data indicate that KmAdhs prefer NAD^+^ and NADH as cofactor. KmAdh1 showed activity in the range of pH 5.0-9.0 when acetaldehyde was the substrate and pH 6.0-9.0 when ethanol was used as the substrate. KmAdh2, KmAdh3 and KmAdh4 showed activities in the range of pH 6.0-10.0. Beyond these pH ranges, the activities of the enzymes were completely lost. The optimum pH of KmAdh1 was measured as 8.0, while those of KmAdh2, KmAdh3 and KmAdh4 were 7.0 (Figure 6a). KmAdh1, KmAdh2, KmAdh3 and KmAdh4 were relatively stable at pH 7.0-9.0, 5.0-9.0, 6.0-9.0 and 6.0-8.0, respectively. They retained more than 60% ADH activity when incubated at the corresponding pH ranges for 24 h (Figure 6b). KmAdh1, KmAdh2 and KmAdh4 showed significant activities between the temperatures of 35 and 55°C, while KmAdh3 showed significant activity between 35 and 65°C. The optimum temperatures of KmAdh1, KmAdh2, KmAdh3 and KmAdh4 were 50, 45, 55 and 45°C, respectively (Figure 6c). KmAdh1 and KmAdh3 were relatively stable in the temperature range of 30 to 45°C, while KmAdh2 and KmAdh4 were relatively stable at 30 to 40°C. The ADH activities decreased sharply above 45°C and were completely lost above 60°C (Figure 6d).

The kinetic properties of the KmAdhs were determined and are summarized in Table 1. The *K*_m_ values of KmAdh1 and KmAdh2 for ethanol were about 10- and 42-fold higher, respectively, than those for acetaldehyde, while the *V*_max_ values for acetaldehyde were about 2- and 12-fold higher, respectively, than those for ethanol. The turnover numbers (*K*_cat_) of KmAdh1 and KmAdh2 for acetaldehyde were 2- and 12-fold and the catalytic efficiencies (*K*_cat_/*K*_m_) were 20- and 520-fold higher than those for ethanol, respectively. These results indicate that KmAdh1 and KmAdh2 of GX-UN120 are chiefly responsible for the reduction of acetaldehyde to ethanol. KmAdh3 and KmAdh4 catalyze the oxidation reaction of ethanol to acetaldehyde but not the reduction reaction of acetaldehyde to ethanol.

**Table 1:** Summary of enzymatic properties of KmAdhs and ADHs from other yeasts

**ADHs**	**Optimum temperature (°C)**	**Optimum pH**	**Ethanol**			**Acetaldehyde**		
			***K***_**m**_**(mM)**	***K***_**cat**_**(min**^**-1**^**)**	***K***_**cat**_**/*K***_**m**_**(min**^**-1**^ **mM**^**-1**^**)**	***K***_**m**_**(mM)**	***K***_**cat**_**(min**^**-1**^**)**	***K***_**cat**_**/*K***_**m**_**(min**^**-1**^ **mM**^**-1**^**)**
KmAdh1^a^	50	8.0	39.7	8.5 × 10^3^	2.1 × 10^2^	4.0	1.7 × 10^4^	4.3 × 10^3^
KmAdh2^a^	45	7.0	49.5	2.7 × 10^2^	5.4	1.2	3.2 × 10^3^	2.7 × 10^3^
KmAdh3^a^	55	7.0	26.0	2.0 × 10^3^	0.8 × 10^2^	n.a.		
KmAdh4^a^	45	7.0	17.0	9.5 × 10^3^	5.6 × 10^2^	n.a.		
KlAdhI^b^	n.d.	n.d.	27	2.5 × 10^5^	9.3 × 10^3^	1.2	3.6 × 10^5^	3.0 × 10^5^
KlAdhII^b^	n.d.	n.d.	23	2.8 × 10^4^	1.2 × 10^3^	1.7	3.3 × 10^4^	2.0 × 10^4^
KlAdhIII^b,c^	n.d.	n.d.	0.5-2.6	8.2 × 10^4^	3.2 × 10^4^	0.1-2.3	8.6 × 10^5^	8.6 × 10^6^
KlAdhIV^b^	n.d.	n.d.	1.6	1.3 × 10^4^	8.3 × 10^3^	3.1	2.9 × 10^4^	9.0 × 10^3^
SceAdh1^d^	30	7.3	17-24	2.0 × 10^4^	1.2 × 10^3^	1.1-3.4	1.0 × 10^5^	9.3 × 10^4^
SceAdh2^d^	30	7.3	0.8	7.8 × 10^3^	9.6 × 10^3^	0.1	6.2 × 10^4^	6.9 × 10^5^
SceAdh3^d^	30	7.3	12	n.d.	n.d.	0.4	n.d.	n.d.
SpAdh^d^	30	7.3	14	n.d.	n.d.	1.6	n.d.	n.d.
HpAdh1^e^	n.d.	n.d.	0.3	2.1 × 10^5^	8.4 × 10^5^	1.9	2.0 × 10^5^	1.0 × 10^5^
ScbAdh1^f^	n.d.	n.d.	18	2.9 × 10^4^	1.6 × 10^3^	1.1	2.1 × 10^5^	1.9 × 10^5^

^a^Data in this study; ^b^Data from Bozzi *et al.*[26]; ^c^Data from Brisdelli *et al.*[27]; ^d^Data from Ganzhorn *et al.*[28] and Thomson JM *et al.*[22]; ^e^Data from Suwannarangsee *et al.*[21]; ^f^Data from Pal *et al.*[29]. Kinetic parameters of KmADH1 for ethanol and acetaldehyde were determined in the presence of 2 mM NAD^+^ or 0.2 mM NADH. KmAdh, ADH of *K. marxianus*; KlAdh, ADH of *K. lactis*; SceAdh, ADH of *S. cerevisiae*; SpAdh, ADH of *S. pombe*; HpAdh, ADH of *H. polymorpha*; ScbAdh, ADH of *S. carlsbergensis*; n.a., no activity detected; n.d., no data available.

### Substrate specificities of the recombinant KmAdhs

**Figure 7:**
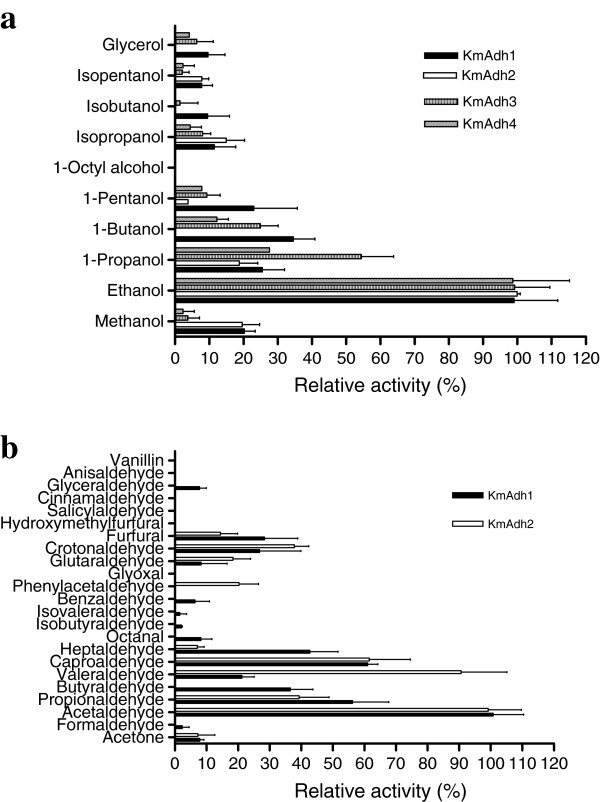
**The activities of the KmAdhs on various alcohols and aldehydes. a** Relative activities on various alcohols; **b** Relative activities on various aldehydes. Experiments were conducted in reaction mixtures containing 50 mM sodium phosphate buffer (pH 7.0), 2 mM NAD^+^ for alcohols or 0.2 mM NADH for aldehydes, 0.8 M alcohols or 50 mM aldehydes and the purified KmAdhs (1–20 μg of protein) at pH 7.0 and 40°C.

The substrate specificities of the recombinant KmAdhs towards different alcohols with various chain lengths were determined and the results are shown in Figure 7a. All four KmAdhs preferred ethanol as the best alcoholic substrate. KmAdh1 and KmAdh2 displayed high activities towards primary 1–5 carbon and 1–3 carbon alcohols, respectively, and the activities decreased with increasing chain length. Both enzymes displayed low or no activity for long chain and branched alcohols. KmAdh3 and KmAdh4 displayed high activities towards ethanol and 1-propanol and low activities for other alcohols. The substrate specificities of the KmAdhs towards 16 straight- and branched-chain aliphatic aldehydes with various chain lengths and 6 aromatic aldehydes were determined and the results are shown in Figure 7b. The highest reducing activities of KmAdh1 and KmAdh2 were found with acetaldehyde. KmAdh1 displayed high activities towards most of the straight-chain aliphatic aldehydes and low or no activity towards the branched-chain aliphatic aldehydes and aromatic aldehydes. The substrate specificity of KmAdh2 towards aldehydes was similar to that of KmAdh1. But the substrate specificities of these two ADHs were different when using butyraldehyde, valeraldehyde, heptaldehyde and phenylacetaldehyde as substrates. Regarding KmAdh3 and KmAdh4, no activities were detected towards any of the tested aldehydes.

## Discussion

The mechanism by which *K. marxianus* produces ethanol at high temperature is unknown as yet. Reports about the ethanol metabolic pathway of *K. marxianus* are rare. In particular, the biochemical characteristics of the ADHs from *K. marxianus*, which contribute to ethanol metabolism, are not understood. The growth and ethanol fermentation characteristics suggest that the fermentation capability of *K. marxianus* GX-UN120 at 40°С is the same as that of *S. cerevisiae* Angel at 34°С. In the present study, all four ADH-encoding genes of GX-UN120 were cloned and overexpressed in *E. coli*. The biochemical characteristics of the purified recombinant KmAdhs were investigated. To our knowledge, this is the first report of the heterologous expression of genes encoding the ADHs of *K. marxianus*.

Amino acid sequence analysis suggests that KmAdh1 and KmAdh2 of GX-UN120 may be cytoplasmic ADHs, while KmAdh3 and KmAdh4 may be mitochondrial ADHs. All four ADHs belong to the microbial group I ADHs. Characterization of their enzymatic properties showed that KmAdhs prefer NAD^+^ and NADH to NADP^+^ and NADPH as cofactor, which is similar to ADHs of other yeasts [25,26,30]. With optimum temperatures of 45-55°C for ethanol and acetaldehyde, the KmAdhs are distinctly different from most reported ADHs of yeasts, which generally have optimum activities at about 30°C [28]. Perhaps this is why GX-UN120 produces its maximal yield of ethanol at 40°C, while other yeasts such as *S. cerevisiae* and *S. carlsbergensis* have maximal yields usually at 30°C [4,19].

There have been no previous reports regarding the substrate specificity of ADHs from *K. marxianus*. Our data indicate that the four recombinant KmAdhs of GX-UN120 have a narrow alcoholic substrate specificity, which is similar to ScAdh1 of *S. cerevisiae*. It was reported that the narrow substrate specificity of ScAdh1 is due to Met^271^ in its substrate binding cleft, whereas there is a Leu in the corresponding position in other yeast ADHs including KmAdhs [14,31]. The alcoholic substrate specificity of the KmAdhs is similar to that of ScAdh1 but different from that of ScAdh2 [14]. The ADHs of *K. lactis*[26,27], Adh1 of *H. polymorpha*[21], ADHs of *C. maltosa*[30] and Adh1 of *C. utilis*[25] display broad alcoholic substrate specificity. KmAdh1 and KmAdh2 of GX-UN120 have a broad substrate specificity for straight-chain aliphatic aldehydes, and the specific activities towards aldehydes are more than 2-fold higher than those towards the analogous alcohols. These results suggest that KmAdh1 and KmAdh2 prefer aldehydes as their substrates and acetaldehyde was the best substrate, which is similar to ADH1s from other yeasts and KlAdh3 [21,26,27,30]. Interestingly, KmAdh1 and KmAdh2 of GX-UN120 could efficiently reduce furfural, which is formed in the pretreatment of lignocelluloses and is an inhibitor of ethanol production by *S. cerevisiae*. This suggests that GX-UN120 is suitable for use in the SSCF of lignocelluloses to produce ethanol.

## Conclusions

Zymogram analysis showed that KmAdh1 was largely induced in *K. marxianus* GX-UN120 during ethanol production, KmAdh4 was constitutively expressed at a lower level and KmAdh2 and KmAdh3 were almost undetectable. The genes encoding the four alcohol dehydrogenases were cloned from strain GX-UN120 and heterologous expressed in *Escherichia coli*. The biochemical characteristics of the recombinant ADHs in this study indicate that KmAdh1 is the primary ADH responsible for the production of ethanol from the reduction of acetaldehyde in *K. marxianus*. The result that the optimum temperature of KmAdh1 was 20°C higher than that of ADH from *S. cerevisiae* may partially explain the ability of *K. marxianus* to produce ethanol at high temperature.

## Methods

### Strains and growth conditions

*K. marxianus* GX-UN120 was used in this study and grown in yeast extract, peptone, dextrose (YPD) medium at 37°С. GX-UN120 is a mutant strain that was derived from the wild-type strain GX-15 which was isolated from soil sample collected in the subtropical area of Guangxi Zhuang Autonomous Region, China [19] and stored in College of Life Science and Technology, Guangxi University, Nanning, China. *E. coli* DH5α (Novagen, USA) and Rosetta DE3 (Novagen, USA) strains were used as the hosts for cloning genes and overexpression of recombinant genes and were grown in LB medium with 100 mg/L of ampicillin at 37°С.

### Growth and ethanol fermentation of *K. marxianus* and *S. cerevisiae*

The growth and ethanol fermentation characteristics of GX-UN120 and *S. cerevisiae* Angel which was obtained from Angel Yeast Co., Ltd, Yichang, China were investigated in 100 mL YPD medium containing 20 g/L glucose in 250-mL Erlenmeyer flasks or 200 mL YPD medium containing 150 g/L glucose in 500-mL Erlenmeyer flasks. The flasks were incubated without shaking. Growth was measured at OD_600_ and the ethanol and glucose concentrations were determined by gas chromatography (GC) and high performance liquid chromatography (HPLC), respectively [19].

### Cloning of genes encoding KmADH from *K. marxianus* GX-UN120

The genomic DNA of GX-UN120 was extracted by the standard method [32] and used as the PCR template. The primers for *KmADH1* and *KmADH2* were designed based on the ADH gene sequences of *K. marxianus* ATCC 12424, and those for *KmADH3* and *KmADH4* were based on the ADH gene sequences of DMKU 3–1042 (Table 2). The genes *KmADH1*, *KmADH3* and *KmADH4* were amplified by PCR with sense primers ADH1F, ADF3F and ADH4F containing a *Bam*HI restriction site at the 5′ end and antisense primers ADH1R, ADH3R and ADH4R containing a *Hin*dIII restriction site at the 5′ end, respectively (Table 2). *KmADH2* was amplified with primers ADH2F and ADH2R containing an *Nde* I and an *Xho* I restriction site at the 5′ end, respectively (Table 2). The PCR products were purified and ligated with pMD-19 T vector. The resulting recombinant plasmids were transformed into *E. coli* DH5α competent cells and the confirmed recombinant plasmids containing *KmADH1*, *KmADH2*, *KmADH3* and *KmADH4* were named as pGXKmADH1, pGXKmADH2, pGXKmADH3 and pGXKmADH4, respectively.

**Table 2:** Primers used in this study

**Primer name**	**Primer sequence**
ADH1F	5′-CGC*GGATCC*AACACAATGGCTATTCCAGAAACTC-3′
ADH1R	5′-GGCCC*AAGCTT*TGGAAGTGTCAACGACAATTCTAC-3′
ADH2F	5′-GGAATTC*CATATG*TCTATTCCAACTACTCAAAAGGG-3′
ADH2R	5′-CCA*CTCGAG*TTTGGAAGTGTCAACAAC-3′
ADH3F	5′-CG*GGATCC*ATGCTTAGATTAACTAACGCCAG-3′
ADH3R	5′-GGCCC*AAGCTT*ACATAATAGACTTCTCTTCTTCAAGAG-3′
ADH4F	5′-CG*GGATCC*CCACATTATACTATTAATAAACCAC-3′
ADH4R	5′- GGCCC*AAGCTT*TTAGCATAGCTTAGTTGGACTG-3′

The letters italic represented the restriction sites.

### Expression of *KmADH* genes in *E. coli* and purification of the recombinant proteins

pGXKmADH1, pGXKmADH3, pGXKmADH4 and the expression vector pET-32a(+) were separately digested with *Bam*HI and *Hin*dIII and the target fragments were purified. pGXKmADH2 and pET-30a(+) were separately digested with *Nde* I and *Xho* I and the gene and the vector DNA were recovered. The DNA fragment containing the *KmAdh* genes and the corresponding expression vector were ligated with T4 DNA ligase to form pET-32a(+)-KmADH1, pET-30a(+)-KmADH2, pET-32a(+)-KmADH3 and pET-32a(+)-KmADH4. The recombinant plasmids were transformed into *E. coli* Rosetta DE3 to express the target proteins. KmAdh1, KmAdh3 and KmAdh4 were expressed as TrxA fusion proteins [33] and KmAdh2 as a His-tagged protein. The recombinant fusion proteins were purified by co-affinity chromatography using a TALON Cobalt Resin column. The purified, recombinant, fusion KmAdhs were digested with enterokinase light chain to remove the TrxA or His-tag. The digestion mixture was loaded on the TALON Cobalt Resin column. The eluted solution containing KmAdh was collected and used for further study, and the TrxA or His-tag bound to the cobalt resin remained in the column. The native molecular masses of the purified KmAdh proteins were measured by high performance gel permeation chromatography (HPGPC) using a Macrosphere GPC 1 507 μ column (250 mm × 4.6 mm, Alltech Associates, Inc.) and eluted with 0.15 M NaCl at a flow rate of 0.3 mL/min. The molecular masses were then calculated using the protein molecular weight standards ferritin horse (450 kDa), catalase bovine (240 kDa), aldolase rabbit (160 kDa) and albumin bovine (67 kDa) (SERVA Electrophoresis GmbH, Heidelberg, Germany).

### Enzyme assay

The zymogram analysis of ADH isozymes and the ADH activities were assayed according to the method previously described by Cho and Jeffries [34] with minor modification. The reaction mixture contained 50 mM sodium phosphate buffer (pH 7.0), 2 mM NAD^+^ or 0.2 mM NADH, 0.8 M alcoholic substrates or 50 mM aldehydic substrates and the purified KmAdhs. One enzyme unit (U) was defined as the micromoles of NADH produced or consumed per minute.

### Kinetic analysis

Kinetic analysis of the KmAdhs was performed as previously described [27]. The *K*_m_ and *V*_max_ values were measured using the double-reciprocal plot method of Lineweaver-Burk [35]. Catalytic efficiency (*k*_cat_/*K*_m_) was derived from *V*_max_/*K*_m_ [*E*].

### DNA sequence analysis

Sequence assembly and ORF analysis were carried out using the Vector NTI program. The protein sequence similarity searches were performed with the BLAST tools (http://www.ncbi.nlm.nih.gov). Only proteins that showed significant similarity and had already been characterized as ADH were used for phylogenetic analysis and multiple sequence alignment. The nucleotide sequences of *KmADH1*, *KmADH2*, *KmADH3* and *KmADH4* were deposited in the GenBank database under accession numbers KF678864, KF678866, KF678865, KF678867, respectively.

## Competing interests

The authors declare that they have no competing interests.

## Authors’ contributions

JJL participated in the design of the study, performed all the experiments, analysed the data and wrote the manuscript. MLZ participated in the research of KmAdh3. MD participated in the research of KmAdh4. ZMM participated in the research of KmAdh1. SXW participated in the research of KmAdh2. YD participated in the research of the growth and ethanol fermentation of the strain. JXF participated in the design of the study and commented on the manuscript. All authors read and approved the final manuscript.

## Supplementary Material

Additional file 1: Table S1 The alcohol dehydrogenase sequences used in this study. Click here for file
