# Supplementary material for: Alcohol dehydrogenases from Kluyveromyces marxianus: heterologous expression in Escherichia coli and biochemical characterization
Source: BMC Biotechnol. 2014 May 21;14:45. doi: 10.1186/1472-6750-14-45 (PMC4062290; doi:10.1186/1472-6750-14-45)
Supplement: Additional file 1: Table S1 — The alcohol dehydrogenase sequences used in this study. [file 1472-6750-14-45-S1.pdf]

## Supplementary materials

### Table S1 The alcohol dehydrogenase sequences used in this study

| Protein name | Source organism | GenBank accession No. | References |
|---|---|---|---|
| KmAdh1 | *Kluyveromyces marxianus* | A1llA4 | [1] |
| KlAdh1 | *Kluyveromyces lactis* | P20369 | [2] |
| KlAdh2 | *Kluyveromyces lactis* | P49383 | [2] |
| KlAdh3 | *Kluyveromyces lactis* | P49384 | [2] |
| KlAdh4 | *Kluyveromyces lactis* | P49385 | [2] |
| KwAdh1 | *Kluyveromyces wickerhamii* | Q6XQ81 | [3] |
| KwAdh3 | *Kluyveromyces wickerhamii* | Q6XQ80 | [3] |
| KwAdh4 | *Kluyveromyces wickerhamii* | Q6XQ79 | [3] |
| ScAdh1 | *Saccharomyces cerevisiae* | P00330 | [4] |
| ScAdh2 | *Saccharomyces cerevisiae* | P00331 | [4] |
| ScAdh3 | *Saccharomyces cerevisiae* | P07246 | [4] |
| ScbAdh1 | *Saccharomyces carlsbergensis* | B6UQD0 | [5] |
| SbAdh1 | *Saccharomyces bayanus* | Q6XQ78 | [3] |
| SbAdh2 | *Saccharomyces bayanus* | Q6XQ77 | [3] |
| SbAdh3 | *Saccharomyces bayanus* | Q6XQ76 | [3] |
| SbAdh5 | *Saccharomyces bayanus* | Q6XQ75 | [3] |
| SkAdh1 | *Saccharomyces kluyveri* | Q6XQ74 | [3] |
| SkAdh2 | *Saccharomyces kluyveri* | Q6XQ73 | [3] |

| | | | |
|---|---|---|---|
| SkAdh3 | *Saccharomyces kluyveri* | Q6XQ72 | [3] |
| SkAdh4 | *Saccharomyces kluyveri* | Q6XQ71 | [3] |
| SpAdh1 | *Saccharomyces pastorianus* | Q6XQ70 | [3] |
| SpAdh2 | *Saccharomyces pastorianus* | Q6XQ69 | [3] |
| SpAdh3 | *Saccharomyces pastorianus* | Q6XQ68 | [3] |
| SpAdh5 | *Saccharomyces pastorianus* | Q6XQ67 | [3] |
| CbAdh1 | *Candida boidinii* | Q75UM7 | [6] |
| CbAdh2 | *Candida boidinii* | Q75UM6 | [6] |
| CbAdh3 | *Candida boidinii* | Q75UM5 | [6] |
| CmAdh1 | *Candida maltosa* | E0XMK3 | [7] |
| CmAdh2A | *Candida maltosa* | E0XMK7 | [7] |
| CmAdh2B | *Candida maltosa* | E0XMK8 | [7] |
| PsAdh1 | *Pichia stipitis* | Q13309 | [8] |
| PaAdh1 | *Pichia angusta* | E0YMC0 | [9] |

## References


1. Ladrière JM, Delcour J, Vandenhaute J: **Sequence of a gene coding for a cytoplasmic alcohol dehydrogenase from *Kluyveromyces marxianus* ATCC 12424.** *Biochim Biophys Acta* 1993, **1173:**99-101.

2. Bozzi A, Saliola M, Falcone C, Bossa F, Martini F: **Structural and biochemical studies of alcohol dehydrogenase isozymes from *Kluyveromyces lactis*.** *Biochim Biophys Acta* 1997, **1339:**133-142.

3. Thomson JM, Gaucher EA, Burgan MF, De Kee DW, Li T, Aris JP, Benner SA: **Resurrecting ancestral alcohol dehydrogenases from yeast.** *Nat Genet* 2005, **37:**630-635.

4. Ganzhorn AJ, Green DW, Hershey AD, Gould RM, Plapp BV: **Kinetic characterization of yeast alcohol dehydrogenases. Amino acid residue 294 and substrate specificity.** *J Biol Chem* 1987, **262:**3754-3761.

5. Pal S, Park DH, Plapp BV: **Activity of yeast alcohol dehydrogenases on benzyl alcohols and benzaldehydes: characterization of ADH1 from *Saccharomyces carlsbergensis* and transition state analysis.** *Chem Biol Interact* 2009, **178:**16-23.

6. Yurimoto H, Lee B, Yasuda F, Sakai Y, Kato N: **Alcohol dehydrogenases that catalyse methyl formate synthesis participate in formaldehyde detoxification in the methylotrophic yeast *Candida boidinii*.** *Yeast* 2004, **21:**341-350.

7. Lin Y, He P, Wang Q, Lu D, Li Z, Wu C, Jiang N: **The alcohol dehydrogenase system in the xylose-fermenting yeast *Candida maltosa*.** *PLoS One* 2010, **5:**e11752.

8. Passoth V, Schaefer B, Liebel B, Weierstall T, Klinner U: **Molecular cloning of alcohol dehydrogenase genes of the yeast *Pichia stipitis* and identification of the fermentative ADH.** *Yeast* 1998, **14:**1311-1325.

9. Suwannarangsee S, Oh D-B, Seo J-W, Kim CH, Rhee SK, Kang HA, Chulalaksananukul W, Kwon O: **Characterization of alcohol dehydrogenase 1 of the thermotolerant methylotrophic yeast *Hansenula polymorpha*.** *Appl Microbiol Biotechnol* 2010, **88:**497-507.
